# Allelopathic and intraspecific growth competition effects establishment of direct sown *Miscanthus*

**DOI:** 10.1111/gcbb.12680

**Published:** 2020-03-30

**Authors:** Danny Awty‐Carroll, Barbara Hauck, John Clifton‐Brown, Paul Robson

**Affiliations:** ^1^ Institute of Biological, Environmental and Rural Sciences Aberystwyth University Gogerddan UK

**Keywords:** agronomy, allelochemical, allelopathic, direct sowing, establishment, *Miscanthus*, *Miscanthus sinensis*, oversowing, seed biology, seed germination

## Abstract

High yielding perennial crops are being developed as a sustainable feedstock for renewable energy and bioproducts. *Miscanthus* is a leading biomass crop, but most plantations comprise a sterile hybrid *Miscanthus* × *giganteus* that is clonally propagated. To develop new varieties across large areas, rhizome cloning is inefficient, time consuming and expensive. Alternative approaches use seed, and in temperate regions, this has been successfully applied by raising seedlings as plug plants in glasshouses before transfer to the field. Direct sowing has yet to be proven commercially viable because poor germination has resulted in inconsistent stand establishment. Oversowing using seed clusters is a common approach to improve the establishment of crops and it was hypothesized that such an approach will improve uniformity of density in early *Miscanthus* stands and thereby improve yield. Sowing multiple seeds creates potential for new interactions, and we identified at least two inhibitory mechanisms related to seed numbers. Germinating seed produced allelopathic effects on nearby seed thereby inhibiting plant growth. The inhibitory effect of *Miscanthus* seed on germination percentages was related to seed number within clusters. An extract from germinating *Miscanthus* seed inhibited the germination of *Miscanthus* seed. The extract was analysed by HPLC, which identified a complex mixture including several known allelopathic compounds including proanthocyanidins and vanillic acid. There was also evidence of root competition in soil in a controlled environment experiment. When the experiment on competition was replicated at field scale, the establishment rates were much lower and there was evidence of shoot competition. We conclude that the numbers of seed required to ensure an acceptable level of establishment in the field may be economically impractical until other agronomic techniques are included either to reduce the inhibitory effects of higher seed numbers or to reduce oversowing rates.

## Graphical abstract

Oversowing *Miscanthus* seed clusters was tested as a means to improve crop establishment. Germinating seed produced allelopathic effects on nearby seed thereby inhibiting plant growth. An extract from germinating *Miscanthus* seed inhibited germination of *Miscanthus* seed. The extract was analysed by HPLC, which identified a complex mixture including several known allelopathic compounds. There was also evidence of root competition in soil in a controlled environment experiment. We conclude the numbers of seed required to ensure an acceptable level of establishment in the field may be economically impractical until other agronomic techniques are included either to reduce the inhibitory effects of higher seed numbers or to reduce oversowing rates.

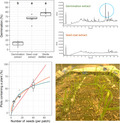

## 1 INTRODUCTION

When *Miscanthus* is planted from rhizome, planting gaps occur due to low rhizome viability and poor establishment (Anderson, Lee, Allen, & Voigt, 2015; Caslin, Finnan, & Easson, 2010; Clifton‐Brown, Breuer, & Jones, 2007). In a perennial crop gaps at establishment influence yield for the lifespan of the crop which for *Miscanthus* may be up to 20 years or more (Hastings et al., 2017). Replanting is often not a commercially viable option and different plant ages may create an unbalanced intraspecific competition resulting in subsequent loss of the introduced plant. Therefore, it is critical to establish a complete and uniform field from the outset.

As an alternative propagation method, direct sowing is half the price of planting by rhizome (Hastings et al., 2017) and genotypes can be scaled quickly via seed to provide a lot of plants (Clifton‐Brown et al., 2017). *Miscanthus* seed has a high thermal requirement for germination (Clifton‐Brown et al., 2011) and without high temperatures on wet soils produces poor crop establishment leading Anderson et al. (2015) to suggest that it was impossible to direct sow *Miscanthus* in much of northern Europe. Switchgrass (*Panicum virgatum*) is often oversown (400 seeds per m^2^) to account for low establishment (Vogel, 1987) and this technique may be suitable to provide a more consistent density of *Miscanthus* plants from seed. In the United Kingdom, direct sowing of *Miscanthus* seed was tested by sowing in rows at 300 seeds per meter (Ashman, Awty‐Carroll, Mos, Robson, & Clifton‐Brown, 2018), but this resulted in uneven emergence. A refinement of this method, that better controls plant density, is oversowing seed in small patches of 1 cm diameter (here referred to as clusters).

As *Miscanthus* develops more into a seed‐based crop (Clifton‐Brown et al., 2017) understanding what effects germination and early seedling growth will be of increasing importance. Seed propagation may be successful if high density sowing will overcome germination and establishment problems. We tested how many seeds per cluster need to be sown to produce an established plant, and if seed competition adversely affects seedling germination and establishment. This is relevant not only to the direct sowing of seeds in the field in particular but also the sowing of seed in tray modules for plug planting. Propagation via plug planting is currently the main method used to establish *Miscanthus* seed‐based hybrids (Clifton‐Brown et al., 2017) and often more than one seed is sown to ensure at least one seedling establishes per plug.

When the *Miscanthus* crop is in the establishment growth phase, plants could be growing very close together. Competition for root space during establishment may determine which plants successfully overwinter. *Miscanthus* plants exhibit separate root systems with dominant plants extending their root systems into a larger territory (de Kroon, Mommer, & Nishiwaki, 2003). This may have an effect on root growth during establishment as the plants expand sideways below ground, while above ground upwards is the primary direction of growth.

### 1.1 Allelopathy and allelochemical effects

Allelochemical effects between plants can be positive or negative (Hussain et al., 2007) and these effects are often used to reduce competition as in the novel weapons hypothesis where exotic invaders take advantage of native susceptibility to allelopathy (Bais, Vepachedu, Gilroy, Callaway, & Vivanco, 2003). Allelopathy may involve interspecific competition or autotoxicity, which would negatively impact a plant's own progeny but may be useful to ensure spatiotemporal distribution of seedling establishment (Ervin & Wetzel, 2000). For instance, Ervin and Wetzel (2000) found autotoxicity to seedlings from tissues of the common rush (*Juncus effusus*), and Giant Parramatta grass (*Sporobolus fertilis*) has been observed to inhibit its seedlings as well as those of other species, but this was not proven in germination tests (Andrews, Jones, & Whalley, 1997).

There is a diverse range of chemicals involved in allelopathic effects that have been identified in different plant species. In wheat (*Triticum aestivum*), Siddiqui, Bhardwaj, Khan, and Meghvanshi (2009) found potential allelochemicals such as flavonoids, waxes, tannins and phenolic acids inhibited seedling growth. A range of alkaloids from mesquite (*Prosopis juliflora*) leaves have been shown to act as growth inhibitors in many species including grasses (Nakano, 2010). Bais et al. (2003) found catechin (a flavonoid)‐induced interspecific allelopathy in *Centaurea maculosa*. Chou and Chung (1974) found that in clearings between bunches of *Miscanthus*, most seedlings were *Miscanthus*. They found acetic acid leached out of the stems and roots into the soil around *Miscanthus floridulus* and affected growth in lettuce, they also found low concentrations of coumaric, ferulic, hydroxybenzoic, syringic and vanillic acids (that did not give significant inhibitory results; Chou & Chung, 1974).

## 2 MATERIALS AND METHODS

Testing of the effects of seed clusters on germination and growth began with laboratory tests of seed in water, then soil testing in a controlled environment and finally field observations. All tests used Japanese derived *Miscanthus sinensis* seed that had a relatively high germination percentage; this was collected from open pollinated *M. sinensis* growing in the United Kingdom (Aberystwyth, Wales).

### 2.1 Testing for allelopathy in water

To test if an allelopathic effect was present in germinating *M. sinensis*, a replicated laboratory experiment was conducted using 12 × 100 mm square Petri dishes with 25 compartments (5 × 5). Each compartment of each dish was filled with 2 ml of sterile distilled water. In 24 of the 25 compartments in replicate each dish, one *M. sinensis* seed was placed, and in the one remaining compartment, 24 *M. sinensis* seeds were placed. The position of the 24 seed compartment in each of the 12 dishes was assigned at random using R, so each replicate dish had both 24 individual seeds and 24 clustered seeds. The *M. sinensis* seed was not sterilized because sterilizing the seed might remove allelopathic chemicals in the seed coat, and because field‐sown seed is unlikely to be sterilized. Dishes were kept in a Fisons Fitotron 600h 120 plant growth chamber (Loughborough, UK) at 25°C with constant fluorescent light at 300 μmol m^−2^ s^−1^ measured with an SKP 215 sensor (Skye Instruments). Germination percentage in the clustered 24 seeds versus the single 24 seeds was observed every 24 hr for 96 hr in each of the 12 dishes.

All data analysis was performed with R (R Core Team, 2015). The results of all time points were analysed with a repeated measures ANOVA, the residuals where checked to be following a normal distribution. Germination index (GI), a combination of germination speed and number (Ranal & de Santana, 2006; Walker‐Simmons, 1987) was used to highlight differences in germination over the whole experiment.

Two further experiments tested at what seed quantity an inhibitory effect of seed occurred. Firstly, a ‘broad range’ experiment, using a 25 compartment 100 mm^2^ Petri dish. This experiment had five replicates of each seed cluster size: 5, 10, 15, 25 and 50 unsterilized *M. sinensis* seeds, these seed cluster numbers were randomly assigned to compartments in one dish. Secondly, a ‘narrow range’ test was used to provide more data on the observed critical threshold of less than 24 seeds. This narrow range test used five replicates set up in the same way, but the numbers of seeds per group were 15, 17, 19, 21 and 23. Germinating seeds were counted every 24 hr for 72 hr. The increased resolution in the second part of the test was to identify if there was a specific concentration of seeds per ml that caused a change in germination inhibition. Statistically the groups at each time point were tested with a one‐way ANOVA and if significant and normally distributed, a Tukey's HSD was conducted to test if there were a specific number of seeds per cluster where the proportion germinating changed.

### 2.2 Testing the seed germination water

In order to determine if the seed coat was the source of allelopathy or if biological activity associated with germination was required for allelopathy, two solutions were produced. The first was germination water made from germinating seed in four dishes (4 × 25), each well had 20 seeds and 2 ml of water, which resulted in 150 ml of seed water due to some absorption/evaporation. The seeds were pre‐sterilized using 100% ethanol for 5 s then rinsed in sterile distilled water, to prevent mould and maximize the number of usable wells. The second extraction was 100 ml of extract from 1,000 non‐germinating (biologically inactive) seeds (in 3 × 33 ml vessels). The seeds were kept at 5°C in sterile distilled water, to prevent germination but allow leaching of seed coat chemicals while preventing germination.

The germinating seed and extract preparations were used as a germination medium to test their effects on *M. sinensis* seed germination. Individual seed was placed in 96 well plates, and each well was randomly assigned to one of three treatment groups, each of 32 wells. The position of the treatments was randomly assigned using R. Each of the three treatment groups received either 0.2 ml of one of the two extracts or, as a control treatment, sterile distilled water. This was replicated five times with a new randomization for each 96 well plate. Seed was germinated for 9 days at 25°C and germination assessed at 9 days to allow for any differences in the speed of germination to even out. A one‐way ANOVA was performed on the germination percentages of each treatment across the five replicates.

Liquid chromatography‐tandem mass spectrometry (LC‐MS*^n^*) was used to identify chemicals in the two extracts, either the seed coat extract or the germinating seed extract. Prior to analysis, extracts were partially purified and concentrated by solid‐phase extraction on 500 mg Sep‐Pak C_18_ 3 cc Vac RC cartridges (Waters Ltd.). Cartridges were preconditioned with 5 ml 100% methanol followed by 5 ml water. Then, 4 ml of sample was loaded, and the cartridges were washed with 3 ml water. Extracts were then eluted with 4 ml 100% methanol, dried down at 50°C under vacuum and re‐suspended in 400 µl 70% MeOH.

LC‐MS analysis was performed on a Thermo Finnigan system (Thermo Electron Corp.) equipped with a Finnigan LTQ linear ion trap with ESI source and a Waters C_18_ Nova‐Pak column (3.9 mm × 150 mm i.d., 4 µm particle size). The autosampler tray temperature was maintained at 5°C and the column temperature at 30°C. The mobile phase consisted of water/0.1% formic acid (solvent A) and MeOH/0.1% formic acid (solvent B). Sample injection volume was typically 25 µl, and the flow rate was 1 ml/min, with 10% of the sample going to the mass spectrometer. The column was equilibrated with 98% solvent A, and the percentage of B increased linearly to 100% over 30 min. Full scan MS data were acquired in negative and positive ionization mode with the following parameters: sheath gas 30, auxiliary gas 15 and sweep gas zero (arbitrary units), spray voltage −4.0 kV in negative and 4.8 kV in positive ionization mode, capillary temperature 320°C, capillary voltage −1.0 and 45 V, respectively, and tube lens voltage −68 and 110 V respectively. MS*^n^* fragmentation was carried out with normalized collision energy of 35% and isolation width *m*/*z* 2.0.

### 2.3 Seed competition soil experiment within a controlled environment

Germination in soil within a controlled environment was used to determine the effect of competition using a more realistic substrate but controlling for other potentially confounding factors such as temperature. This experiment used 3 mm sieved and autoclaved soil collected from the field described in Ashman et al. (2018; Aberystwyth, Wales, 52°24′53.8″N, 004°02′31.7″W). Four replicate trays (360 × 210 mm) were filled 50 mm deep with a sandy loam soil, then 32 (4 × 8) divots were made (15 mm × 15 mm × 15 mm) in the surface of each replicate tray 30 mm apart. Into the central 1 cm^2^ of each divot was placed 1, 5, 15 or 40 *M. sinensis* seeds allocated randomly to one of the four lines of eight divots, resulting in a randomized complete block design. Results were averaged within each set of eight divots for each replicate tray. Each tray was covered with SAMCO grey mulch film (Adare, Ireland) as previously described for use in *Miscanthus* field planting (Ashman et al., 2018). The trays were placed in a line in a Fisons Fitotron 600h 120 plant growth chamber, and kept under constant illumination (from both the tungsten and fluorescent lighting 170 ± 10 µmol m^−2^ s^−1^; Caffarra, Donnelly, Chuine, and Jones, 2011), at 25°C for 32 days. The environment in the cabinet was measured using a ‘Campbell 1000’ data logger with two type T thermocouples (trays 2 & 4) and two reflectometers (trays 1 & 3), soil moisture was maintained at (35%–40% w/v), this was higher than the real field conditions.

Germination/tillering and elongation of tallest stem per cluster of seeds sown in situ were recorded on days 2, 4, 7, 10, 14, 22 and 32. On day 22, the film was removed; this was because the seedlings had grown beyond the limits of the tray, and in the field, the film would be beginning to degrade at this point (without sun the photodegradable film was not degrading in the cabinet). On day 32, the seedling clusters were removed and elongation of the tallest stem in each cluster was measured. This process was carried out both for total elongation to the end of the leaf and height to the youngest ligule. The length of the longest root in the whole seedling cluster was recorded as a measure of below ground growth. From the roots, the number of individual plants was counted, to get an accurate final germination score and a score of tillering. The seedling clusters were dried at 70°C for 48 hr to determine dry weights. Then, the roots were removed and the root and shoot dry weights were determined to calculate the proportion of biomass above and below ground.

The germination was statistically tested over the first 7 days with a generalized linear mixed‐effects model using a binomial distribution (‘lme4’ R package; Bates, Mächler, Bolker, & Walker 2015). This was then checked with a one‐way ANOVA, by testing the change in tillers between 48 and 168 hr; differences were grouped using a Tukey's HSD. The number of plants counted at the end of the experiment was used to determine the number of seedlings per seed sown and the impact of seed number tested by one‐way ANOVA.

The impact of below ground competition on root size when grown in clusters of different seed numbers was tested in a number of ways. The overall dry weight of roots per seedling cluster at the end of the test was log^10^ transformed to produce a normal distribution and analysed with a one‐way ANOVA. The dry weight of roots was divided by the number of seeds sown and tested separately. Maximum elongation of roots in each seedling cluster and the maximum elongation per plant calculated. The effect of seed number on root phenotypes was analysed by one‐way ANOVA and if effects were significant, further analysed using Tukey's HSD.

A linear mixed‐effects model (R ‘nlme’ package; Pinheiro, Bates, DebRoy, Sarkar, & R Core Team, 2016) was used to determine if there was a positive or negative effect of cluster size on elongation rate during the experiment. Finally, elongation, height, total tillers, stem dry weight and overall dry weight at the end of the experiment were each analysed with a one‐way ANOVA, for the average per height, tiller number etc. within each plot and the average per plant in the plot. This was because some metrics, such as total number of stems/tillers, are likely to be higher the more seeds sown; therefore, it was more relevant to consider the effect per plant.

### 2.4 Field trial of cluster sowing seed

Cluster sowing was tested over 1 year in a replicated field trial. This experiment was designed to investigate longer term growth, and survival over a winter, and assess the chance of a successful plant at each location in field conditions where germination is often low. A field near Aberystwyth (52°41′41.1″N, 4°04′17.2″W) was prepared by spraying with Roundup^TM^, then, the soil tilth was improved with a power harrow. Seed from the same *M. sinensis* seed lot was used in the field trial as was used in the controlled environment experiments. Seed was sown directly into soil by hand in clusters of 5, 15 and 40. Single seed clusters were not used because the germination percentage in the field was expected to be lower than in the controlled environment experiments; therefore, it was anticipated that so few seed would germinate that most or all plots of single seed would lack a plant. The field design was of four randomized replicate blocks, each block consisting of two rows (covered by a single piece of SAMCO mulch film) containing 45 clusters each. Thirty of each of the three different cluster sizes (5, 15 and 40 seeds) were placed randomly within the 90 cluster locations of seed per block. Each seed cluster was approximately 1 cm in diameter and clusters were spaced at 0.5 m × 0.5 m, interblock spacing was 1.1 m to allow for a path.

Hand weeding was required intermittently during the summer growth season. Commercially weed control would likely have been carried out with a broad leaf herbicide (e.g. Calisto^TM^), but the effect of herbicides on post emergent *Miscanthus* was unknown and it was decided not to risk affecting the crop (Anderson, Voigt, Bollero, & Hager, 2010). The trial was harvested at the normal harvest time for *Miscanthus* of early spring (March 2016). At the end of the trial, the number of plants per cluster was counted as well as the number of tillers; it was not possible to measure the height of the tillers due to winter senescence, which resulted in a number of snapped tillers. Consequently, measuring the effect of sowing density on yield as reported by Vogel (1987) was not possible. The above and below ground growth from each seed cluster was harvested and washed to remove soil. For each cluster, the length of the longest root was measured, and plants were counted by picking apart the cluster. The samples were then dried (70°C for 48 hr) and the below ground dry weight of biomass determined.

Firstly, the data were analysed to determine if the number of seed sown in a cluster affected the presence or absence of a plant. Output from this analysis was used to determine how many seeds need to be sown for a successful plant, and if the number of plants in a plot was proportional to the number of seed sown. To analyse the number of plants per cluster relative to the cluster size, a Pearson's correlation was calculated and tested for significance. This was done both using the total number of plants and the number of plants per seed sown to get a relative value for each cluster. The proportion of successful plots was used to predict the number of seed required per cluster to achieve a 100% successful plots; this was estimated using several regression models (linear, natural log, square root) fitted through the origin to show the difference in fit and prediction of each.

Secondly, the data were analysed to determine if root growth was affected by the cluster size. This was done for the total per cluster size and the average per plant within each cluster size, in order to determine if there was an effect independent of the number of surviving plants after winter. This was tested using an ANOVA with a blocking factor on both the root dry weight and the root length data, if significance was detected, a Tukey's HSD test was used to determine what cluster size had the effect.

The root dry weights from all individual seed clusters were correlated against the number of plants counted in every cluster to test if at the plot level, the roots' mass increased linearly to the number of plants present in the plot. This would be expected if there was no adverse effect on below ground growth from more plants in a plot.

Finally, to determine how the above ground growth was affected by the number of seed sown per cluster, the mean number of stems per seed sown and the mean number of tillers per plant were tested with an ANOVA with a blocking factor, if there was a significant effect, a Tukey's HSD estimated the groupings. The average number of stems per cluster was correlated with the number of seed sown using a Pearson's product moment correlation.

## 3 RESULTS

### 3.1 Testing seed for allelopathy in water

**Figure 1:**
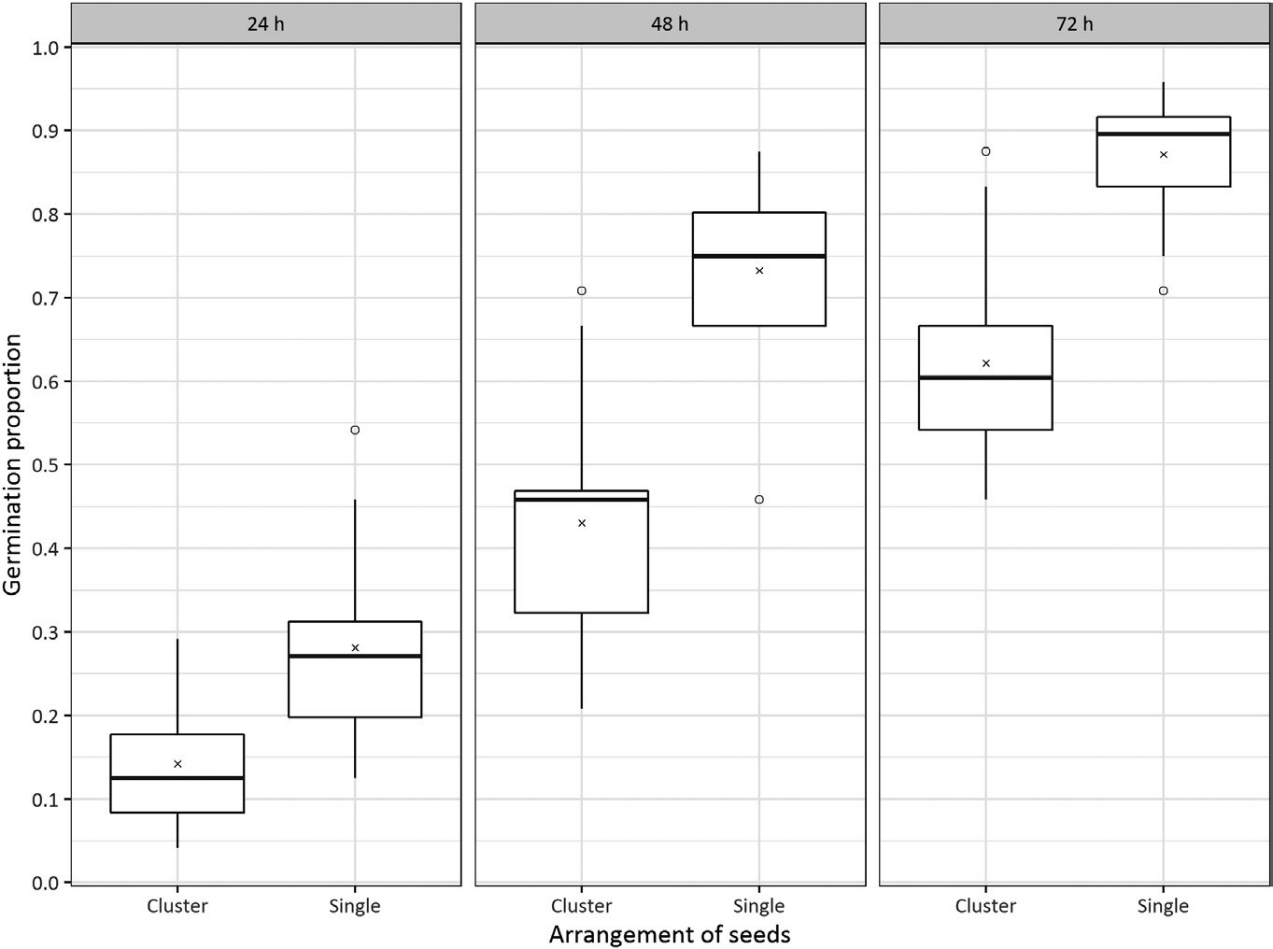
Boxplot of the proportion of *Miscanthus sinensis* seed germinated at 24, 48, 72 and 96 hr at 25°C, comparing germination rates from clusters of 24 seeds and 24 single seeds. Each boxplot represents the distribution across 12 replicates; the mean is indicated by an × symbol on each box

The number of seeds in a cluster had a significant effect on the proportion of germinated seed (*p* < .001); at 72 hr, the clustered seed germination was reduced by 25 ± 3% *SE* (Figure 1). The GI was calculated from the means of each of two seed cluster sizes over time. The GI of single seed was 3.18, and for the clusters of seeds 1.91. There was mould observed in this test, particularly in the compartments with many seeds.

**Figure 2:**
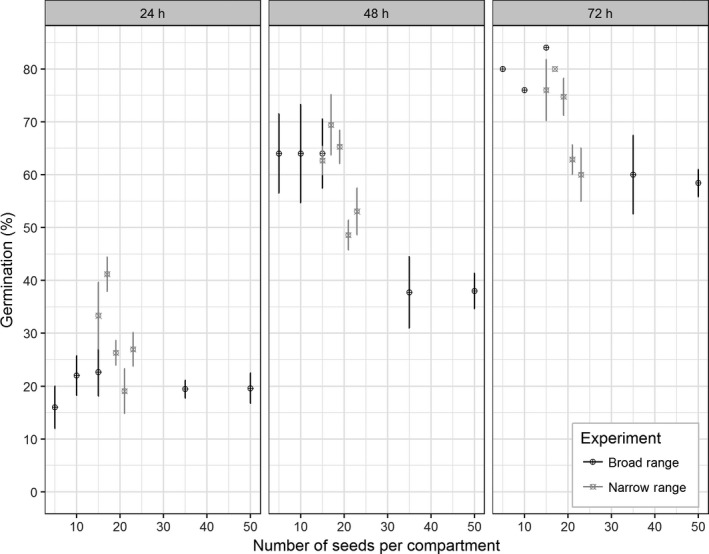
The percentage of seed germinating when sown in clusters of varying size. Two tests are shown: the broad range test from 5 to 50 seeds (black symbols), and the narrow range test from 15 to 23 seeds (grey symbols). The data are shown separately for 24, 48 and 72 hr of incubation at 25°C. The error bar represents the standard error, *n* = 5

Phenotypes were grouped into either high or low percentage germination when seed clusters varying in size from 5 to 50 seeds were examined (Figure 2). A broad range test was used to identify approximately how many seeds resulted in decreased germination, and then, a narrow range test was used to identify more accurately the seed number associated with this decline.

In the broad range test (Figure 2 [Black]), after 48 and 72 hr, the clusters containing less than 20 seeds were germinating to a higher percentage than clusters of more than 20 seeds. The germination rate in seed clusters of 5–15 seeds was an average of 80% at 72 hr; this rate fell to only 60% germination for larger seed clusters of 35 and 50 seeds.

The narrow range test (light grey symbols in Figure 2) of 15–23 seeds identified germination rates in the higher and lower categories that were consistent with the broad range experiment. When a sigmoid curve was fitted to the data from both these tests at 72 hr, the midpoint between the two groups was located at 19.89 seeds. Therefore, the impact on germination percentage occurred at ~20 seeds, clusters with less than 20 seeds germinated on average at 77% at 72 hr, and clusters with more than 20 seeds germinated at ~61% at 72 hr. In the broad range test, the number of seeds per cluster had a significant (*p* < .05) effect on germination at 72 hr. The narrow range test had a more gradual yet still significant (*p* < .05) change in germination percentage (Figure 2). These results suggest that there is a cut‐off point for the number of seeds in a cluster, after which germination decreased by ~20%.

### 3.2 The impact on germination of water extracts of seed and germinating seed

**Figure 3:**
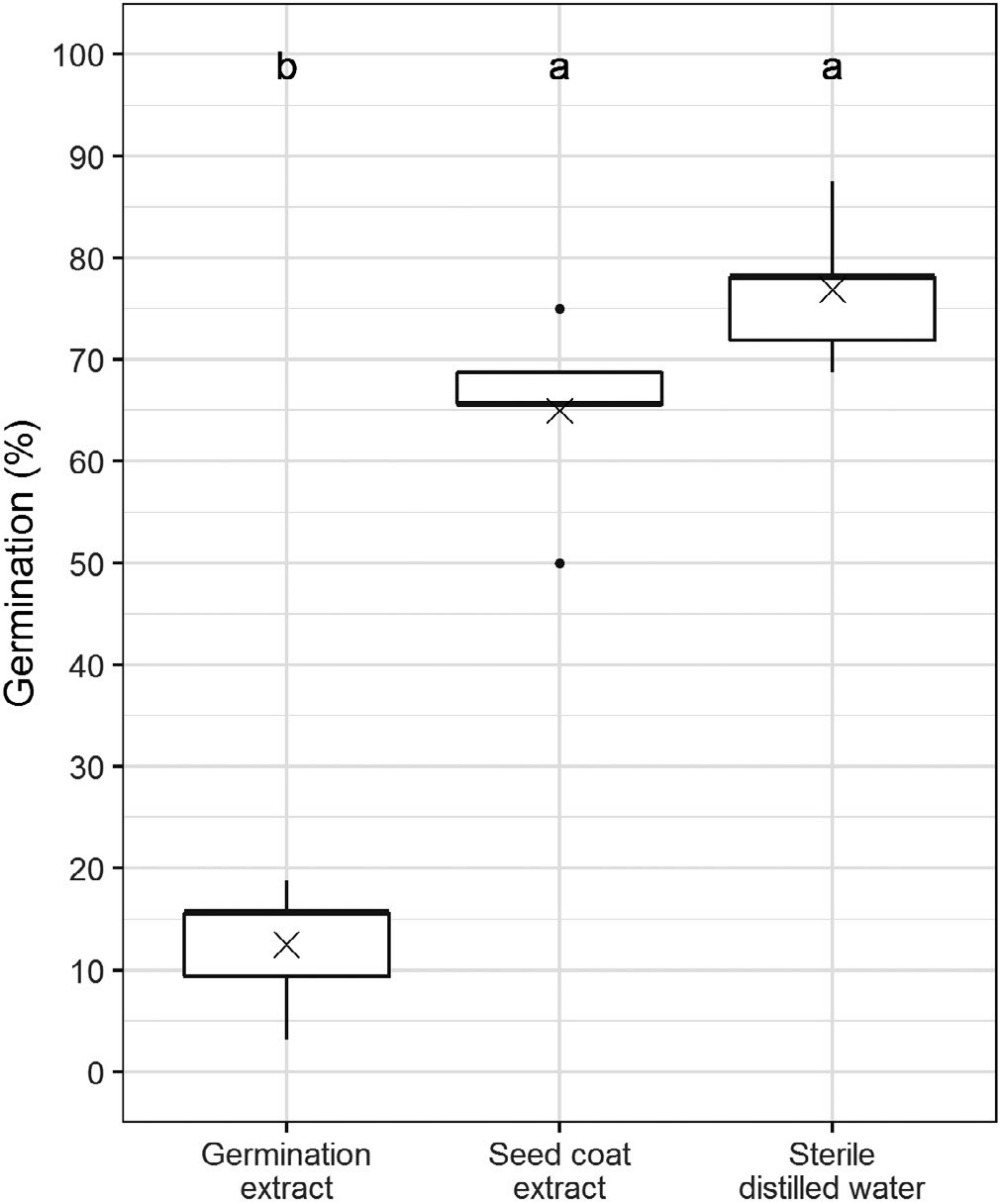
The percentage of seed germinated after 9 days of incubation at 25°C in two solutions extracted from either germinating or non‐germinating seed and in sterile distilled water as a control. Each boxplot represents the distribution across five replicates; the mean is indicated by an × symbol on each box. Letters indicate significant groupings by Tukey's HSD

When seeds were soaked in water at lower temperatures to inhibit germination, the resulting solution (seed coat extract) did not significantly effect germination rates compared with SDW (Figure 3). When seeds were germinated in water, the resulting solution (germination extract) had a significant effect on germination of seed compared with SDW (*p* < .001).

### 3.3 Seed competition in soil within a controlled environment

All seed clusters sown into soil in a controlled environment cabinet produced at least one plant except single seed, which had an average success rate of 59%. The average total number of plants per cluster (as counted at the end of the experiment) was significantly (*p* < .0001) higher when more seeds were sown (Table 1). Clusters formed four significant groupings, the highest number of plants corresponded to the cluster with the highest seed number.

**Table 1:** Growth analysis from clusters of different seed numbers in soil under controlled environment conditions

Seeds sown	No. of plants	Dry mass (mg)	Stem mass (mg)	Root mass (mg)	Root length (cm)
Per cluster					
1	0.6 ± 0.03	6.7 ± 1.4	5.2 ± 1	1.5 ± 0.6	11.4 ± 3.6
5	3.4 ± 0.29	36.8 ± 9.9	29.7 ± 7.5	7.0 ± 2.5	36.5 ± 7.7
15	8.4 ± 0.36	73.4 ± 21.7	62.3 ± 17.4	11.1 ± 4.4	47.9 ± 13.0
40	20.9 ± 1	136.4 ± 21.3	115.7 ± 18.6	20.7 ± 2.7	45.6 ± 9.3
Per seed					
1	0.59 ± 0.03	6.71 ± 1.38	5.2 ± 0.95	1.15 ± 0.59	11.4 ± 3.6
5	0.68 ± 0.06	7.35 ± 1.98	5.94 ± 1.5	1.41 ± 0.5	7.3 ± 1.5
15	0.56 ± 0.02	4.89 ± 1.45	4.15 ± 1.16	0.74 ± 0.29	3.2 ± 0.9
40	0.52 ± 0.02	3.41 ± 0.35	2.89 ± 0.47	0.52 ± 0.07	1.1 ± 0.2

Note

Results are expressed first as the average of four replicates, and secondly as the average divided by the number of seeds sown in the cluster.

All numbers are shown with the standard error (*n* = 4).

The number of plants per seed sown appeared to be effected by cluster size (Table 1), but this was not significant (*p* = .053). Clusters sown with five seeds produced the highest number of plants per seed sown, but this effect was not statistically significant (Table 1).

Root dry weight was significantly affected by cluster size (*p* < .01) which increased with increasing cluster size (Table 1). Clusters were in two significant groupings; 1 and 5 seeds were in the lower weight group with 5, 15 and 40 seeds in the higher weight group. The effect on dry weight of roots per seed sown was not significant (*p* = .11; Table 1).

The average maximum root length per replicate was 11.4 ± 3.6 mm for single seeds, 36.5 ± 7.7 mm for five seed clusters, 47.9 ± 13 mm for 15 seed clusters and 45.6 ± 9.3 mm for 40 seed clusters (±*SE*, *n* = 4). This was not significantly affected by cluster size (*p* = .08); however, the length of seedlings per seed sown was significantly affected (*p* < .0001). The clusters had three groupings; the longest roots were from 1 to 5 seeds, then 15 and the shortest roots from 40 seeds per seed cluster.

**Figure 4:**
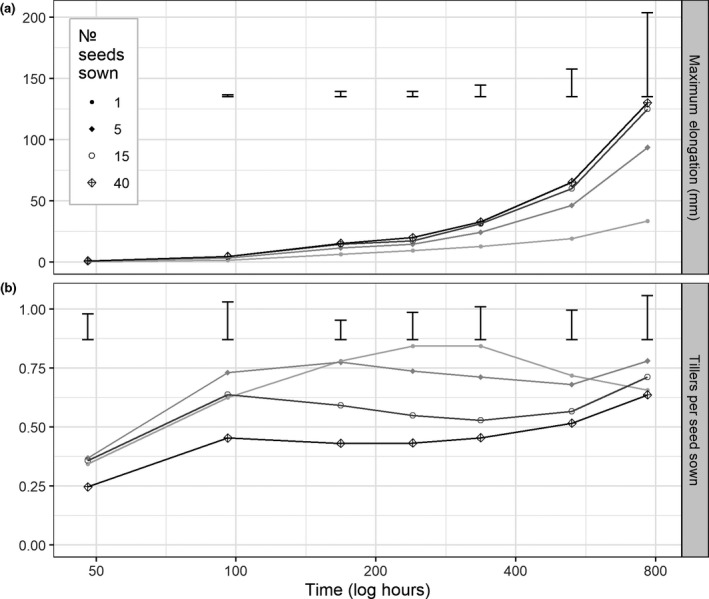
The number of tillers per seed sown (b) and the maximum above‐ground elongation growth of seed clusters (a) sown in soil in a controlled environment cabinet. Means are within each replicate (*n* = 4) for each cluster size. The darkness of the line increases with increasing number of seed sown in the cluster. Error bars denote the least significant difference between seed numbers at each time point (0.05)

Stem elongation measured over the course of the controlled environment experiment (Figure 4a) was analysed with a linear mixed‐effect model using the position in the controlled environment as the random effect. This showed a significant effect on stem elongation from time as the plants grow but not cluster size (*p* < .001, *p* = .59).

For the final measurements at the end of the experiment, the averaged heights were significantly (*p* < .01) affected by the number of seed per cluster. Clusters with 5, 15 and 40 seeds produced a group of significantly taller (~22 to 35 cm) plants while clusters of 1 and 5 seeds produced shorter plants (~8 to 22 cm). When height was analysed for effect per seed sown, there was a significant (*p* < .0001) effect of cluster size. Clusters containing 1 and 5 seeds were the tallest averaging ~6 cm per seed sown, then 5 and 15 seeds averaged ~3 cm and 15 and 40 seeds per cluster were significantly shorter averaging ~1 cm.

Figure 4b shows the number of tillers per cluster, this was significantly affected by the number of seeds sown (*p* < .0001). Clusters of 40 seeds produced the most tillers (~26), then 15 (~11) and lowest 5 and 1 seed clusters (~4 and 1 respectively). However, the number of tillers per plant was not significantly affected by cluster size (*p* = .13).

The dry weight of stems was significantly affected by cluster size (*p* < .0001). This produced three groupings; the highest mass of 116 mg was in the 40 seed clusters, then an intermediate group of the 5 and 15 seed clusters (~46 mg), as well as a low mass group (~18 mg) of the 1 and 5 seed clusters (Table 1). Stem dry weights per plant were not significantly affected by the number of seed sown in the cluster (*p* = .1; Table 1).

The number of seeds sown significantly affected the total dry weight per cluster (*p* < .0001). This was formed of three significant groups: 15 and 40 (~105 mg), 5 and 15 (~55 mg) and 1 and 5 seeds per cluster (~22 mg; Table 1). However, total dry weight per established plant was also not significantly effected by cluster size (*p* = .09; Table 1).

### 3.4 Seed competition in soil under field conditions

Clusters of 5, 15 and 40 seeds growing under field conditions were harvested at the end of the first growth year. The mean number of plants growing in the clusters at the end of the year (Table 2) increased approximately linearly with the number of seeds in the cluster and was highly correlated (Pearson's product moment correlation coefficient = 0.89, *p* < .001).

**Table 2:** Morphology after one growth year resulting from different cluster sizes of seed sown in UK field conditions

Seeds sown	No. of plants	No. of stems	Root mass (mg)	Root length (cm)
Per cluster				
5	0.3 ± 0.1	0.6 ± 0.1	53.1 ± 12.7	8.9 ± 1.4
15	0.8 ± 0.1	1.7 ± 0.4	82.9 ± 14.2	9.0 ± 1.1
40	1.9 ± 0.3	3.2 ± 0.7	98.4 ± 17.6	9.8 ± 1.2
Per seed				
5	0.06 ± 0.01	0.13 ± 0.03	10.62 ± 2.53	1.77 ± 1.4
15	0.05 ± 0.00	0.11 ± 0.02	5.53 ± 0.95	0.6 ± 1.1
40	0.05 ± 0.00	0.08 ± 0.02	2.46 ± 2.46	0.2 ± 1.2

Note

Shown first as the average for the replicate, and secondly as the average divided by the number of seeds sown. All numbers are shown with the standard error within the four replicates.

The number of plants per seed sown (Table 2) was highest at five seeds (~0.06 plants per seed sown) and stable for 15–40 seeds (~0.05 plants per seed sown). There was a weak negative correlation between the number of seed in a cluster and the number of plants per seed sown (*r* = −.35), but this was not significant (*p* = .31).

**Figure 5:**
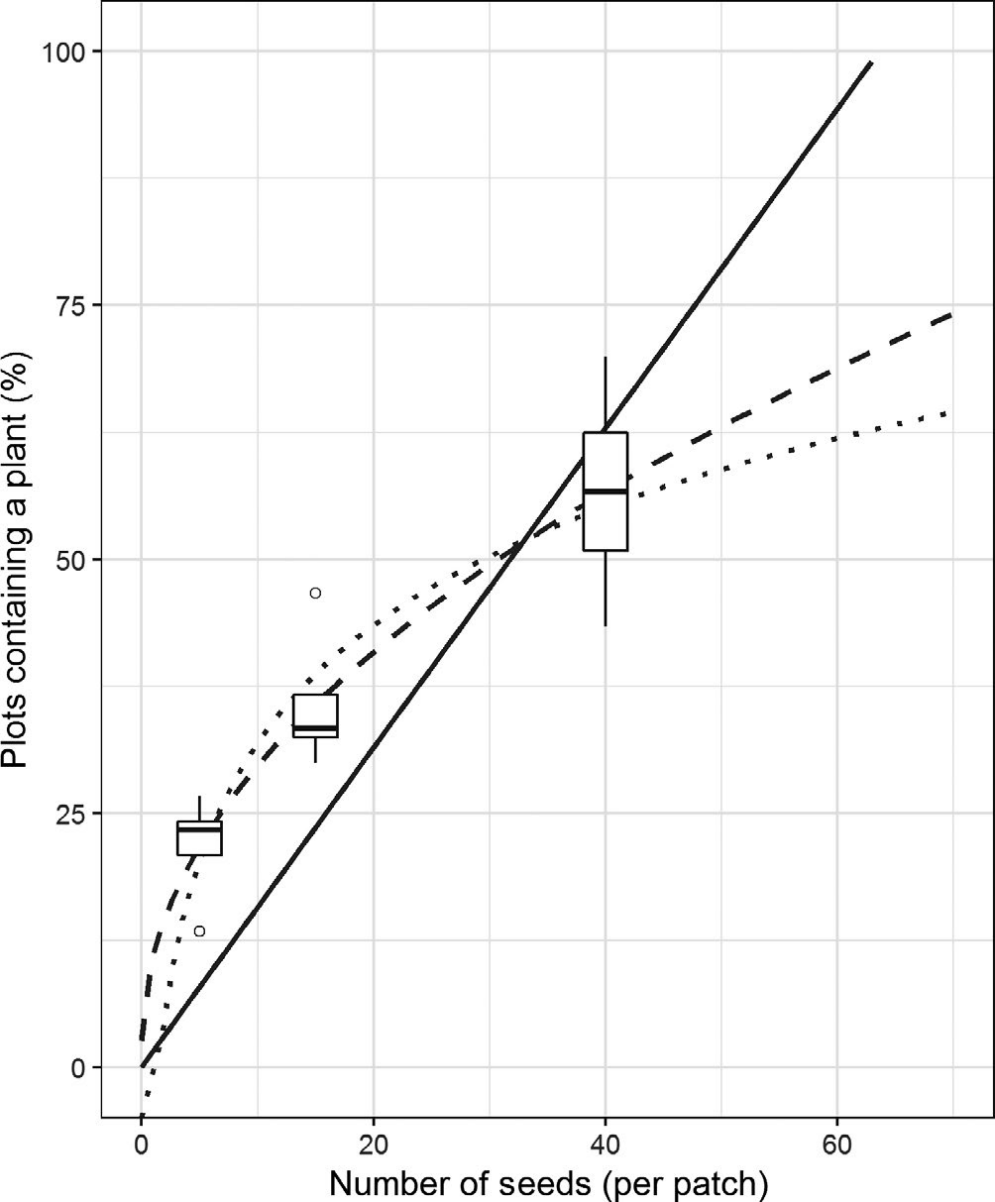
Estimating the proportion of sown clusters with at least one plant surviving after 10 months in the UK field. Three models, a linear model through the origin (solid line), a natural log model (dotted line) and a square root model (dashed line), were fit to the data (*n* = 4). The models were extended to 70 seeds per cluster, to estimate the chance of at least one plant per plot

The chance of a plant surviving in a plot significantly (*p* < .001) improved as the number of seeds per cluster increased (Figure 5). However, all four replicates are well below 100% of clusters producing a plant. Three models were tested to estimate how many seeds would be required to achieve ~100% of clusters containing a plant (Figure 5).

Three fitted models were used to predict the cluster size required to produce at least one plant per cluster. The linear model, with an *R* ^2^ of .31, suggested ~64 seeds per cluster would be required for the average plot to produce an ~100% success rate of one plant per cluster (Figure 5, solid line). The natural log model (Figure 5, dotted line) produced a better approximation with an *R* ^2^ of .78; however, using this model 64 seeds would result in a success rate of 63%, and clusters of hundreds of seeds would be needed to achieve 100% success rate. The square root model (Figure 5, dashed) fitted the data most accurately with an *R* ^2^ of .79 and indicated that 137 seeds would be required per cluster to achieve an ~100% success rate.

The harvested root dry weights showed no significant (*p* = .07) increase in root mass after 1 year when more seeds were sown (as long as the cluster survived). The dry weight of roots per plant was also not effected by number of seeds sown (*p* = .09); there was, however, a significant effect of block (*p* < .01) which may have obfuscated any true effect. There was no significant effect of seed number per cluster on the mean lengths of the longest root per cluster (*p* = .35).

The difference in mean root elongation per plant counted (Table 3) was significantly effected by cluster size (*p* < .05 and blocking significant at <.05). The means grouped into seed clusters of 5 and 15 as an upper grouping and 15 and 40 seeds as a lower grouping.

**Table 3:** The average maximum root length from seed clusters of different sizes sown in the field. Root lengths were averaged per block for each cluster size; the average was also divided by the number of plants in the cluster. Data were collected after 10 months in the UK field. Standard errors were calculated from the four replicates

No. of seeds sown	Average root length per patch	Divided by number of surviving plants
5	8.85 ± 1.41	7.47 ± 1.24
15	9.01 ± 1.11	5.18 ± 0.8
40	9.76 ± 1.23	4.19 ± 0.5

**Figure 6:**
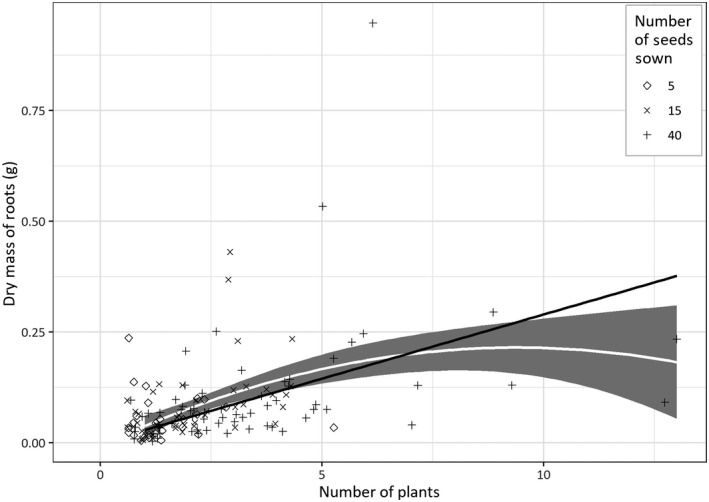
The effect of the number of plants on below ground dry weight for all 360 clusters individually after 10 months in the UK field. Both measurements were taken at the start of the second year (March), just after the time, a mature crop would ordinarily be harvested in the United Kingdom. The position of the points on the *x*‐axis has been randomly offset by up to 0.4 to reduce overplotting. A second‐order polynomial line with 95% confidence interval was added and a linear model fitted through the origin. Both lines have an *R* ^2^ of .44. If root mass was dependent on number of plants, the model will follow a linear trend

Finding competition by testing if dry weight was affected by the number of seeds per cluster ignores the relative success of each cluster. Therefore, Table 3 shows the effect of number of plants growing on the dry mass of all roots for every cluster; this is similar to what is seen in Figure 6, but not averaged within the blocks. The dry weight increases with the number of plants alive per cluster until five plants approx., before levelling off (Table 2). This would be linear if roots were the same regardless of number of plants growing in the cluster.

The number of tillers per plant and a visual assessment of plant health were used to assess above ground growth. Clusters with lots of plants tended to have many weak small plants, and one or two large multi‐tillered plants. For example, a plot with five plants and 10 tillers was much more likely to have a plant with four tillers, two plants with two tillers and three plants with one tiller than to have five plants with two tillers each.

Table 2 gives a strong linear correlation with a Pearson's correlation coefficient of .79 between stems at the end of the trial and the number of seeds per cluster. The mean number of stems per cluster was significantly affected by cluster size (*p* < .01); this produced two groupings, one with 40 and 15 seed clusters and a lower stem number group with 5 and 15 seeds per cluster. This will be primarily due to more seed per cluster producing more plants. There is a slight downwards trend seen in Table 2 for the number of tillers per plant per seed sown, which was not significant (*p* = .13; this had a significant blocking factor with *p* < .05).

### 3.5 Chemical analysis

**Figure 7:**
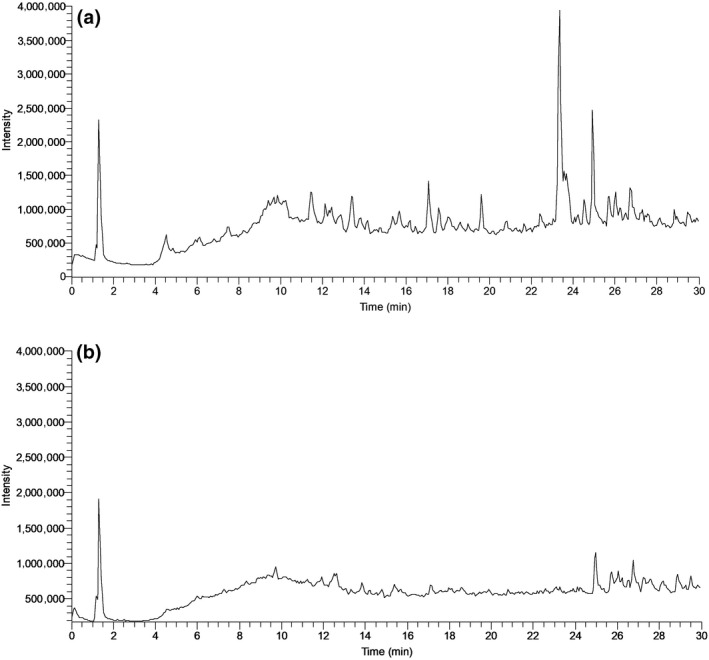
Liquid chromatography‐tandem mass spectrometry chromatograms over *m*/*z* range from 200 to 1,000 in negative mode of seed coat extract (a) and germination extract (b)

**Figure 8:**
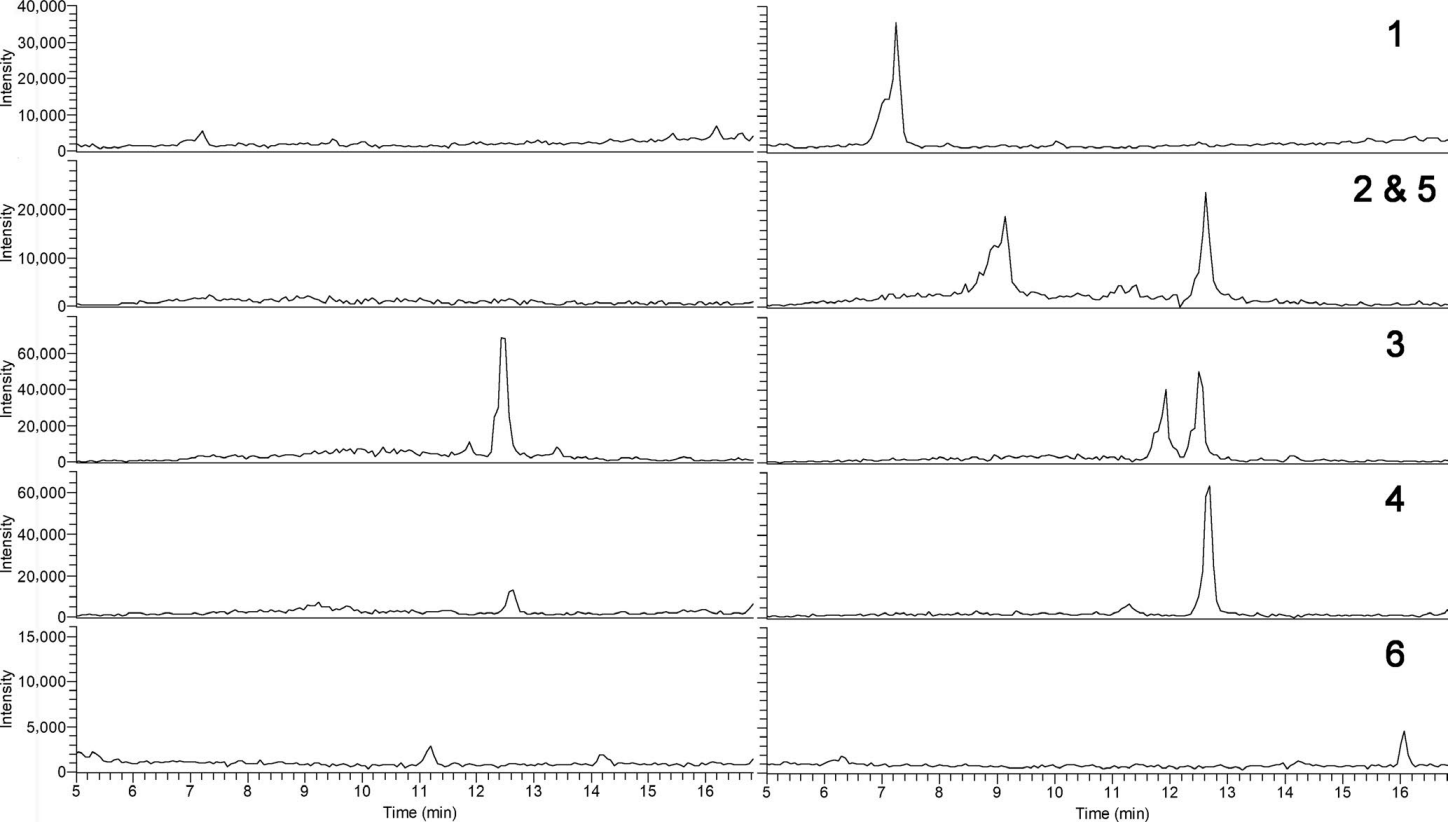
Extracted ion chromatograms in negative mode for compounds 1, 2 and 5, 3, 4 and 6 (top to bottom) in seed coat extract (left column) and germination extract (right column)

Comparison of chromatograms showing *m*/*z* range from 200 to 1,000 in negative ionization mode (Figure 7) revealed complex samples, with more prominent peaks present in seed coat extract. In order to identify compounds which were elevated in germination extract, the chromatogram scan range was split into several narrower mass ranges. The extracted ion chromatograms in negative mode for the compounds shown by this method to be more abundant in germination extract are shown in Figure 8. This included compound 1 which was mostly present as formic adduct and was tentatively identified as vanillic acid hexoside, based on its molecular weight and aglycone fragmentation pattern consistent with vanillic acid standard (Table 4). Compound 4, eluting at 12.6 min, was detected in both ionization modes and was in negative mode also predominantly present as a formic adduct. Tandem mass spectrometry yielded fragments consistent with procyanidin type A dimers (Appeldoorn, Sanders, et al., 2009). Similarly, the molecular weight and fragmentation pattern of compound 5, which was co‐eluting with compound 4, are in agreement with those reported by Gu et al. (2003) and Appeldoorn, Vincken, Sanders, Hollman, and Gruppen (2009) for procyanidin trimers with two A‐type linkages. Compound 2 showed the same nominal mass as compound 4 and neutral losses typical of flavan‐3‐ols and was therefore identified as a putative A‐type proanthocyanidin trimer.

**Table 4:** Compounds with elevated levels in the water extract of germinating *Miscanthus* seed

No.	RT (min)	MW	Formic adduct (*m*/*z*, negative mode)	Characteristic MS*^n^* fragments in negative mode (*m*/*z*, MS^2^ base peak in bold)	Tentative compound identification
1	7.2	330	375	**167**, 152, 123, 108	Vanillic acid hexoside
2	9.1	862		817, **735**, 709, 693, 571, 445	Proanthocyanidin type A trimer
3	11.9	882	926	**745**, 719, 593, 557, 447, 431, 405, 269	Flavanone dimer di‐*O*‐hexoside
4	12.6	576	621	**449**, 423, 407, 289, 285	Procyanidin type A dimer
5	12.6	862		**735**, 709, 575, 571, 449, 289, 285	Procyanidin type A trimer
6	16.0	224		137, **179**, 205	

A compound with MW 882 was present in both seed coat water and germination water at RT 12.5 min. MS^2^ fragmentation products in negative mode included ions with *m*/*z* 719 and 557, corresponding to the neutral loss of two hexose sugars (Vukics & Guttman, 2010). MS*^n^* analysis of the aglycone yielded product ions similar to those reported for Gb‐2a (naringenin‐I,3‐II,8‐eriodictyol; Akpanika et al., 2017). However, as there were small differences in some of the low abundance, characteristic fragments, we tentatively identify this compound as a flavanone dimer. An isomer of this compound with elevated levels in germination water was detected at RT 11.9 min. In addition, an unknown compound with *m*/*z* 223 in negative mode was detected in germination extract at RT 16.0 min.

## 4 DISCUSSION

### 4.1 Seed competition in water

Seed competition was detected when *Miscanthus* seeds were germinated in water suggesting the presence of an intraspecific allelochemical effect. There was a sudden change in germination rate with concentration of seeds; lower numbers of seed (15 or less) showed high germination, and a significant reduction in germination percentage occurred at greater seed numbers. It is likely that whatever activity causes the germination phenotype gains potency over a narrow concentration range. Such responses have been reported for ABA which downregulates germination (Baskin & Baskin, 2004; Finch‐Savage & Leubner‐Metzger, 2006; Shu, Liu, Xie, & He, 2016) and has a steep operation curve (Grappin, Bouinot, Sotta, Miginiac, & Jullien, 2000).

Extracts from *Miscanthus* seed coat and from germinating seed were chemically complex, but several candidate/potential allelochemicals with elevated levels in germinating seed extract were identified by LC‐MS. These include proanthocyanidins, which maintain the levels of ABA to delay seed germination (Jia et al., 2012) and vanillic acid which also lowers germination in weed species (Reigosa, Souto, & Gonźalez, 1999). Furthermore, vanillic acid has been shown to reduce germination in *Sorghum bicolor* (Einhellig & Rasmussen, 1978), a species closely related to *Miscanthus*. However, due to the large number of chemicals in the extracts, definitive identification of the active constituent(s), among many possible allelochemicals (Bais et al., 2003; Siddiqui et al., 2009), was not possible.

### 4.2 Seed competition in soils

After one growth year seed sown as clusters in the field were well established, with larger seedlings in each plot. However, small seedlings were still often surviving within each cluster and therefore potentially competing with the larger plant. There was less noticeable competition from soil‐grown seed clusters in a controlled environment, perhaps because environmental conditions, including availability of water and temperature, were less limiting of growth. This comparison does suggest caution in modelling absolute rates of germination between controlled and field conditions even when the substrate is identical.

Germination of single seed was 41% lower when sown in soil than the expected almost 100% germination rate of the seed lot used; this is probably due to the microclimate around the seed such as temperature, water contact from soil and other inhibitory factors such as the presence of mould. However, seed clusters successfully established complete plots at a high rate under controlled environment conditions. In the field, the chance of achieving one plant per sowing position was low: approximately 24% for five seeds and 50%–60% for 40 seeds. If the success rate was not effected by additional seed sown, approximately 64 seeds would be needed to approach a 100% success rate.

In both field and controlled environment experiments, there was a strong positive correlation between the number of seeds sown and the number of plants produced. If we assume an ideal condition, the number of plants growing increases linearly with the number of seeds sown. The deviation from this linear change was observed as plants per seed sown increased. This deviation was not significant in either environment. In the field, there was a decline (from 4% to 7% in five seeds, versus 4% to 5% in 15 and 40 seeds), whereas in the controlled environment, germination per seed was higher in clusters of five seeds than in individual seeds and clusters of 15 seeds. This may imply that at around 15 seeds per cluster, there is a drop in germination resulting in a lower number of plants. This is similar to the number of seed identified when testing allelopathic effects of different numbers of *Miscanthus* seed germinating in water.

The length of the longest root was independent of the number of seeds sown in the cluster. Root dry weight should increase significantly with number of seeds sown, because more seeds will lead to more plants; however, below ground competition for space could mean all clusters take up approximately the same space. In the controlled environment, below ground dry weight was distributed as expected with more seed resulting in more root dry weight. In the field trial, there was an increase related to cluster size, but this was not significant. In both of the experiments, the decrease in root dry weight per plant as cluster size increased was not significant, but in the field, this change had a shallower slope which may reflect the larger volume available for root growth.

By dividing the length of the longest stem by the number of plants present at the end of the test, it should be possible to detect competition effects between plants even if the competing plants were no longer alive. This would be the case if one or two of the largest plants outcompeted the others within a cluster, but were slowed down by these competing seedlings. However, in both the controlled environment soil and the field tests, the decrease detected with cluster size was not significant.

There was a significant proportional increase in height according to the number of seed sown, and the elongation over time was significant. This could indicate elongation is occurring as a result of intraspecific competition or signalling between seedlings such as shade avoidance (Casal, Sanchez, & Botto, 1998; Pierik & De Wit, 2014). Above ground dry weight significantly increased with the number of seeds sown, as might be expected from more plants resulting in more biomass. However, when divided by the number of plants, there was a non‐significant decrease with number of seeds sown reinforcing the inference of above ground competition.

The numbers of tillers produced by seed clusters significantly increased with seed number in controlled environment and field experiments. The effect of seed number on the mean number of tillers per plant was not significant but acted in opposite directions; with increasing seed sown, the number of tillers per plant increased in controlled environment but decreased in the field. Early increase in tiller number could be an effect of competition to outgrow neighbouring plants, but over time in the field, this competition has resulted in fewer tillers per plant due to tiller abortion or increased apical dominance from shade avoidance responses (Franklin, 2008). The latter may explain the difference between controlled environment and field in that the higher levels of far‐red light in the natural light environment would likely produce a phytochrome photoequilibrium that produced a more significant shade avoidance response (Franklin, 2008; Pierik & De Wit, 2014).

The health of individual plants in the field cluster was observed to be more variable in the clusters with more plants, perhaps as a result of stress from intraspecific competition. However, health did not vary according to the number of seeds sown, the larger plants were healthy and therefore, the effects on plant health were disproportionately acting on the smaller plants.

The laboratory tests showed a clear effect of direct seed competition on germination, but in the field, there will also be the effect from the suboptimal conditions and competition from weeds and weed seeds. With much lower germination in the field, it is not possible to know to what extent the allelopathic inhibition found in the laboratory is preventing germination in larger clusters because of the concerted effects from other factors. Identification and verification of which chemical(s) are contributing to the allelopathic effect will require further study.

Current seeding rates for plug plants require too few seeds per plug to be effected by the allelopathic effects identified in this study. However, plugs may contain small numbers of plants growing in controlled environments where seedlings in our experiments demonstrated positive outcomes from intraspecific competition.

Oversowing will potentially result in intraspecific competition, a potentially negative effect, and larger *Miscanthus* plants appeared to grow more to compete with weaker plants during the first year. Whether this early period of competition results in weaker plants or acts as an eustress producing a more competitive plot requires further investigation. Oversowing increases the likelihood of producing a complete stand without gaps. The interactions of factors, such as those elucidated in the present study, mean the relationship between seed sown and successful establishment was not linear. It is predicted that sowing between 64 and 120 seeds per cluster will produce a complete plot in the field each time if seed demonstrated 100% germination rate seen under controlled conditions. At present, this level of oversowing appears unrealistic but improved understanding of the factors that impact germination and subsequent growth and of the genotypic variation in thermal requirements for germination (Clifton‐Brown et al., 2011) will bring the possibility of direct sowing complete *Miscanthus* stands closer to reality.

## Data Availability

The experimental designs and raw results from this study are available in an OSF repository, osf.io/2qwy8.
